# *Anopheles gambiae* (*s.l*.) exhibit high intensity pyrethroid resistance throughout Southern and Central Mali (2016–2018): PBO or next generation LLINs may provide greater control

**DOI:** 10.1186/s13071-020-04100-7

**Published:** 2020-05-08

**Authors:** Arthur Sovi, Chitan Keita, Youssouf Sinaba, Abdourhamane Dicko, Ibrahim Traore, Moussa B. M. Cisse, Ousmane Koita, Dereje Dengela, Cecilia Flatley, Elie Bankineza, Jules Mihigo, Allison Belemvire, Jenny Carlson, Christen Fornadel, Richard M. Oxborough

**Affiliations:** 1 Faculty of Agronomy, University of Parakou, BP123 Parakou, Benin; 2 Centre de Recherche Entomologique de Cotonou, 06BP2604 Cotonou, Benin; 3 Disease Control Department, Faculty of Infectious & Tropical Diseases, The London School of Hygiene and Tropical Medicine, Keppel Street, London, WC1E 7HT UK; 4 PMI VectorLink Project, Abt Associates, Cite du Niger 1, Rue 30, Porte 612, Bamako, Mali; 5 Programme National de Lutte contre le Paludisme (PNLP), Ministère de la Santé, Bamako, Mali; 6 Université des Sciences, des Techniques, et des Technologies de Bamako (USTTB), Bamako, Mali; 7 PMI VectorLink Project, Abt Associates, 6130 Executive Blvd, Rockville, MD 20852 USA; 8 U.S. President’s Malaria Initiative, U.S. Agency for International Development, Bamako, Mali; 9 U.S. President’s Malaria Initiative, U.S. Agency for International Development, Washington, DC USA; 10 Innovative Vector Control Consortium (IVCC), Washington, D.C USA

**Keywords:** Susceptibility test, Resistance intensity, WHO tube test, CDC bottle bioassay, Piperonyl butoxide, Vector control, Indoor residual spraying, Long-lasting insecticidal net, *Anopheles gambiae*, Mali

## Abstract

**Background:**

Millions of pyrethroid LLINs have been distributed in Mali during the past 20 years which, along with agricultural use, has increased the selection pressure on malaria vector populations. This study investigated pyrethroid resistance intensity and susceptible status of malaria vectors to alternative insecticides to guide choice of insecticides for LLINs and IRS for effective control of malaria vectors.

**Methods:**

For 3 years between 2016 and 2018, susceptibility testing was conducted annually in 14–16 sites covering southern and central Mali. *Anopheles gambiae* (*s.l*.) were collected from larval sites and adult mosquitoes exposed in WHO tube tests to diagnostic doses of bendiocarb (0.1%) and pirimiphos-methyl (0.25%). Resistance intensity tests were conducted using CDC bottle bioassays (2016–2017) and WHO tube tests (2018) at 1×, 2×, 5×, and 10× the diagnostic concentration of permethrin, deltamethrin and alpha-cypermethrin. WHO tube tests were conducted with pre-exposure to the synergist PBO followed by permethrin or deltamethrin. Chlorfenapyr was tested in CDC bottle bioassays at 100 µg active ingredient per bottle and clothianidin at 2% in WHO tube tests. PCR was performed to identify species within the *An. gambiae* complex.

**Results:**

In all sites *An. gambiae* (*s.l*.) showed high intensity resistance to permethrin and deltamethrin in CDC bottle bioassay tests in 2016 and 2017. In 2018, the WHO intensity tests resulted in survivors at all sites for permethrin, deltamethrin and alpha-cypermethrin when tested at 10× the diagnostic dose. Across all sites mean mortality was 33.7% with permethrin (0.75%) compared with 71.8% when pre-exposed to PBO (4%), representing a 2.13-fold increase in mortality. A similar trend was recorded for deltamethrin. There was susceptibility to pirimiphos-methyl, chlorfenapyr and clothianidin in all surveyed sites, including current IRS sites in Mopti Region. *An. coluzzii* was the primary species in 4 of 6 regions.

**Conclusions:**

Widespread high intensity pyrethroid resistance was recorded during 2016–2018 and is likely to compromise the effectiveness of pyrethroid LLINs in Mali. PBO or chlorfenapyr LLINs should provide improved control of *An. gambiae* (*s.l*.). Clothianidin and pirimiphos-methyl insecticides are currently being used for IRS as part of a rotation strategy based on susceptibility being confirmed in this study.
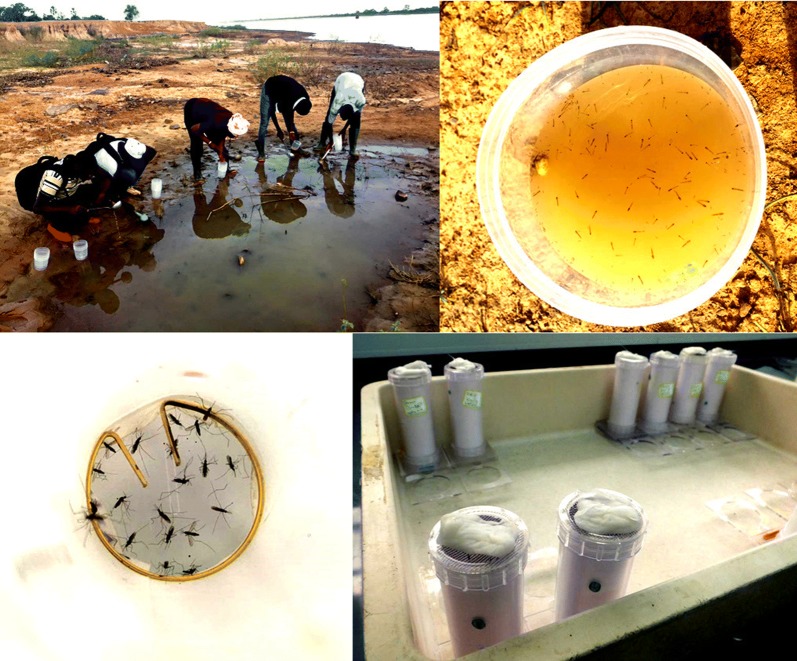

## Background

Malaria remains an important disease in Mali, with the Demographic and Health Survey (DHS) of 2018 estimating the prevalence in children aged 6–59 months to be highest in the regions of Sikasso (30%), Segou (26%), Mopti (25%) and Koulikoro (22%), with Bamako having the lowest prevalence (1%) [1]. The Mali National Malaria Control Programme (NMCP) relies on two methods of malaria vector control, namely nationwide distribution of long-lasting insecticidal nets (LLINs) and targeted indoor residual spraying (IRS). Since 2004, efforts have been made by the Mali NMCP, supported by donors including the U.S. President’s Malaria Initiative (PMI) and The Global Fund, to reach universal coverage of LLINs (1 net for every 2 people) throughout the country [2]. Millions of pyrethroid LLINs have been distributed in Mali during the past 20 years which, along with agricultural use of pyrethroids, has increased the selection pressure on malaria vector populations. Mass LLIN distribution campaigns are conducted on a staggered basis at the regional level approximately every three years, with the most recent mass campaigns in Kayes and Mopti regions in 2017 and Koulikoro and Sikasso regions in 2018. In addition, since 2006 the NMCP has supported free distribution of LLINs to pregnant women during antenatal care and children following vaccination [2]. In 2018 the average number of LLINs owned per household was relatively high in Southern and Central Mali, with an average of 2.2 (Bamako) to 3.0 (Mopti) LLINs per household. Questionnaires as part of the Demographic Health Survey in 2018 showed that 56% of the population in Bamako slept under an LLIN the previous night, while the highest rate was in Mopti Region at 82% [1].

PMI Indoor Residual Spraying (IRS) was conducted annually in selected high malaria burden districts, covering between100,000–250,000 houses per year, since 2008. A pyrethroid insecticide (lambda-cyhalothrin) was used for IRS for three years from 2008–2011, in Bla and Koulikoro districts, with the addition of Baroueli district in 2011. Following the detection of pyrethroid resistance, a carbamate insecticide (bendiocarb WP), was sprayed for four years (2011–2014). Wagman et al. [3] showed that during 2012–2014, rapid reductions in malaria incidence were observed during the six months following each IRS campaign in Segou Region, with an estimated 286,745 total fewer cases of all-age malaria cases observed in IRS districts.

Between 2014 and 2018 a long-lasting organophosphate formulation was sprayed (pirimiphos-methyl CS) annually. According to the Malaria Indicator Survey of 2015, the incidence of malaria in the Mopti region was twice that of the national average [4]. Therefore, IRS operations were relocated to four districts (Djenné, Mopti, Bandiagara and Bankass) of Mopti Region where an organophosphate was sprayed in 2017, and an organophosphate and neonicotinoid were sprayed in 2018.

While the efficacy of LLINs [5] and IRS [6] in reducing malaria transmission is proven beyond doubt, insecticide resistance seriously threatens to jeopardize vector control efforts [7, 8]. A previous study by Cisse et al. in 2012 showed widespread pyrethroid resistance in *Anopheles gambiae* (*s.l*.), the main malaria vector complex in Mali [9]. It is therefore important to regularly monitor the susceptibility level of malaria vectors to insecticides used for vector control to help guide national decision making. This study was performed in Mali for three years between 2016 and 2018 in 14–16 sites nationwide, with the primary aim being to update information on the susceptibility status of *An. gambiae* (*s.l*.) to pyrethroid, carbamate, organophosphate, neonicotinoid and pyrolle insecticides that can be used for malaria control. Additionally, the intensity of resistance to pyrethoids that are used on LLINs and the response to the synergist piperonyl butoxide (PBO) was determined.

## Methods

### Study area

**Fig. 1:**
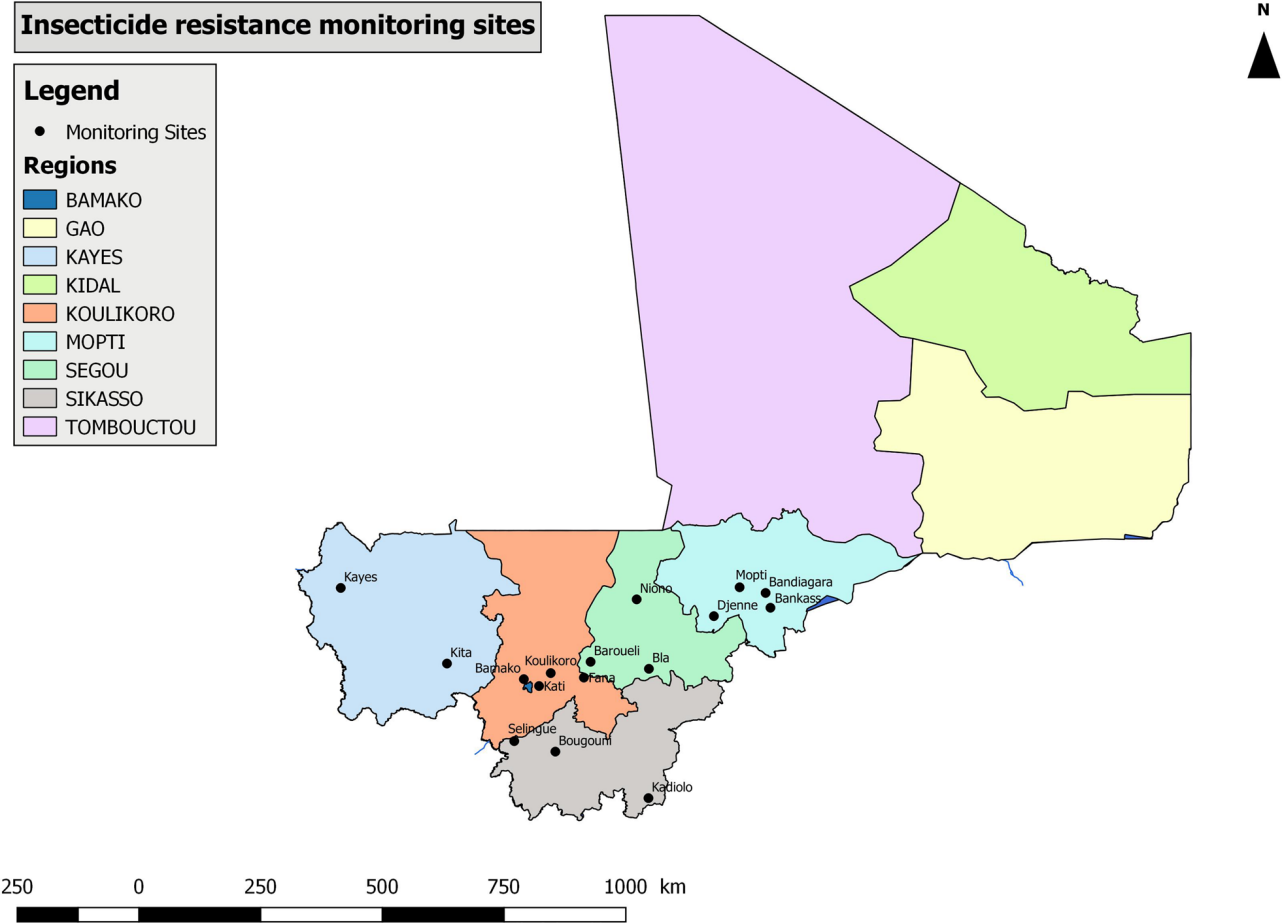
Map showing the insecticide monitoring sites in Mali

The study was conducted in 2016 and 2017 in 16 monitoring sites located within 6 regions covering Southern and Central Mali. In 2018, tests were conducted in 14 sites, with no testing conducted in Fana and Baroueli. No testing was conducted in Northern Mali (Gao, Kidal and Timbuktu regions) due to security concerns. These 16 sites were selected for various reasons, including selection pressure from agriculture and public health vector control (Table 1) and previous use by NMCP for resistance monitoring. By using the same sites annually, resistance trends to insecticides in mosquitoes could be monitored over time. Geographical locations of the 16 sites are shown in Fig. 1.

**Table 1:** Characteristics of surveillance sites used for insecticide resistance monitoring

Region	District	History of insecticide use
Kayes	Kayes	Intense use of insecticides for agriculture. Crops include cotton and ground nuts, sorghum, maize, rice, millet, sweet potatoes, beans and various vegetables.
	Kita	
Koulikoro	Koulikoro	Annual IRS with lambda-cyhalothrin (pyrethroid) 2008–2011, bendiocarb (carbamate) 2011–2014 and pirimiphos-methyl (organophosphate) 2014–2016.
	Fana	Single round of IRS with pirimiphos-methyl (organophosphate) in 2016.
	Kati	Irrigated agriculture including cotton, groundnuts and tobacco. Use of insecticides to control *Simulium damnosum* larvae (blackfly).
Segou	Niono	Large areas of irrigated rice agriculture and pesticide use.
	Bla	IRS with pirimiphos-methyl (organophosphate) in 2014.
	Baroueli	Annual IRS with lambda-cyhalothrin (pyrethroid) 2008–2011, bendiocarb (carbamate) 2011–2014 and pirimiphos-methyl (organophosphate) 2014–2016.
Sikasso	Bougouni	Intense use of insecticides for growing of cotton.
	Sélingué	Irrigated agriculture and pesticide use. Crops include various vegetables and fruits.
	Kadiolo	
Mopti	Bandiangara	IRS with pirimiphos-methyl (organophosphate) in 2017 and 2018.
	Mopti	
	Bankass	
	Djenné	IRS with pirimiphos-methyl (organophosphate) in 2017 and clothianidin (neonicotinoid) in 2018.
Bamako	Bamako	Urban areas where domestic personal protection is used (insecticide aerosols, coils).

### Mosquito larvae collections and rearing

Larval collections of *An. gambiae* (*s.l*.) were conducted annually in the monitoring sites in 2016, 2017 and 2018 from July to October (during the rainy season). Mosquito larvae and pupae were collected from several larval pools in each site, sorted by genus before being grouped together and brought back to the field insectary for rearing into adult mosquitoes. After emerging into adults, mosquitoes were identified to species and only *An. gambiae* (*s.l*.) were tested.

### CDC bottle bioassays

In 2016 and 2017, pyrethroid resistance intensity was determined using Centers for Disease Control and Prevention (CDC) bottle bioassays, which involved coating 250 ml glass bottles with 1×, 2×, 5× and 10× the diagnostic concentration of permethrin (21.5, 43, 107.5 and 215 µg active ingredient (ai)/bottle) and deltamethrin (12.5, 25, 62.5, 125 µg ai/bottle) [10, 11]. For all bottle bioassay tests, a mouth aspirator was used to introduce 20–25 female *An. gambiae* (*s.l*.) aged 3–5 days into each bottle, with four bottles tested per insecticide dose. In 2018, a comparison of the CDC bottle bioassay and the World Health Organization (WHO) tube test for pyrethroid intensity was conducted in two sites using permethrin and deltamethrin.

Chlorfenapyr susceptibility was determined using bottles treated with 100 µg ai/bottle. At the time of testing there was no published guidance regarding chlorfenapyr susceptibility procedures or diagnostic concentrations, while work coordinated by the WHO was ongoing to develop a suitable procedure. The dose of 100 µg ai/bottle was used as an interim diagnostic concentration based on unpublished data shared between the manufacturer, BASF, PMI and CDC. Wild-caught *An. gambiae* (*s.l*.) from Djenné, Mopti, Bandiagara and Bankass were tested for susceptibility to chlorfenapyr. Testing was conducted simultaneously with a susceptible insectary strain (*An. coluzzii* Ngousso).

A bottle coated with 1 ml acetone was used as a control. Vials of permethrin and deltamethrin technical grade active ingredient (TGAI) were provided by CDC at pre-measured concentrations for intensity tests. A vial of chlorfenapyr TGAI was provided by BASF. The diagnostic time was 30 min for pyrethroids, based on CDC guidelines [12]. Chlorfenapyr tests were conducted for the diagnostic time of 60 min, with mortality recorded every 24 h for 72 h.

### WHO susceptibility tube tests

In 2018, pyrethroid resistance intensity was assessed using WHO tube tests with 1×, 5× and 10× the diagnostic concentration of alpha-cypermethrin (0.05%, 0.25% and 0.5%), permethrin (0.75%, 3.75% and 7.5%) and deltamethrin (0.05%, 0.25% and 0.5%). Synergist assays were also conducted by pre-exposing mosquitoes to WHO papers treated with piperonyl butoxide (PBO) (4%) for 60 min before being immediately transferred to a different WHO tube with a pyrethroid treated paper (alpha-cypermethrin (0.05%), permethrin (0.75%), or deltamethrin (0.05%)) for a further 60 min [13]. WHO tube tests were also performed to determine susceptibility status to bendiocarb (0.1%) and pirimiphos-methyl (0.25%) in 2016, 2017 and 2018.

At the time of testing there was no published guidance regarding clothianidin susceptibility procedures or diagnostic concentrations, while work coordinated by the WHO was ongoing to develop a suitable protocol. Therefore, an interim protocol was developed for impregnating filter papers for tube tests. The clothianidin dosage was determined based on internal testing conducted by the manufacturer, Sumitomo Chemical Company (SCC, Tokyo, Japan), which showed that 1% clothianidin active ingredient provided 100% mortality against five insectary strains of *An. gambiae* and *An. arabiensis* [14]. The diagnostic dose was set at 2% weight/volume (w/v) SumiShield™ 50WG (i.e. twice the minimum dose that killed 100%), with distilled water used as a solvent. Clothianidin tests were conducted using filter papers prepared in Mali. A solution was prepared using 264 mg SumiShield™ 50WG dissolved in 20 ml distilled water. Whatman® No.1 filter papers were treated using a pipette to dispense 2 ml of solution on each 12 × 15 cm filter paper, resulting in a concentration of 13.2 mg/ai clothianidin per paper. Clothianidin susceptibility testing was conducted in 2018 in four locations where IRS was conducted, namely Djenné, Mopti, Bandiagara and, Bankass (all in Mopti region).

In all tests four batches of 20–25 non-blood-fed female *An. gambiae* (*s.l*.) adults (field collected as larvae) aged 3–5 days were used for the tube tests according to the WHO protocols [13]. All insecticide impregnated papers (except for clothianidin, which was prepared in situ) were prepared by the WHO collaborating center, Universiti Sains, Malaysia. Knockdown was recorded at the end of the exposure period at 60 min. Mosquitoes were transferred into untreated observation tubes and provided with cotton wool soaked with 10% sugar solution. Mortality was recorded 24 h after exposure for all insecticides, except clothianidin, which was recorded daily for up to 5 days, in order to record any delayed mortality effects. Testing and holding conditions were regulated using a humidifier and air conditioner unit to stay within the WHO limits (temperature of 27 ± 2 °C and a relative humidity of 75 ± 10%).

### Molecular species identification and detection of pyrethroid resistance alleles

In 2017 and 2018, approximately 50 female *An. gambiae* (*s.l*.) from each site were randomly chosen from the untreated controls after the CDC bottle bioassay (2017) and the WHO tube test (2018), and were analyzed by PCR for species identification according to the protocol described by Santolamazza et al. [15]. This method distinguishes between *An. gambiae*, *An. coluzzii* and *An. arabiensis* (members of the *An. gambiae* (*s.l*.) species complex). Using the same mosquitoes, the frequency of the voltage-gated sodium channel (*vgsc*)1014F (previously *kdr* west) and 1014S (previously *kdr* east) mutations was estimated according to the protocols of Martinez-Torres et al. [16] and Ranson et al. [17].

### Data analysis and interpretation

Resistance status of each site was determined according to the WHO criteria [13]:

(i) low resistance intensity (mortality < 90% at 1× diagnostic dose and between 98–100% at 5× dose); (ii) moderate resistance intensity (mortality < 98% at the 5× dose but between 98–100% at the 10× dose); and (iii) high resistance intensity (mortality < 98% at the 10× dose). The frequency of resistance mutations (*vgsc*1014F,1014S) was determined using the formula: F= [(2RR+ RS)] / [2(RR+RS+SS)]. Comparison was made between mortality rates with and without PBO pre-exposure using the Chi-square test as described by Campbell & Richardson [18, 19]. The same methodology was used for comparison of the WHO tube tests and CDC bottle bioassays in Niono and Koulikoro.

## Results

### *An. gambiae* (*s.l*.) intensity of resistance to permethrin and deltamethrin in CDC bottle bioassays in 2016 and 2017

**Fig. 2:**
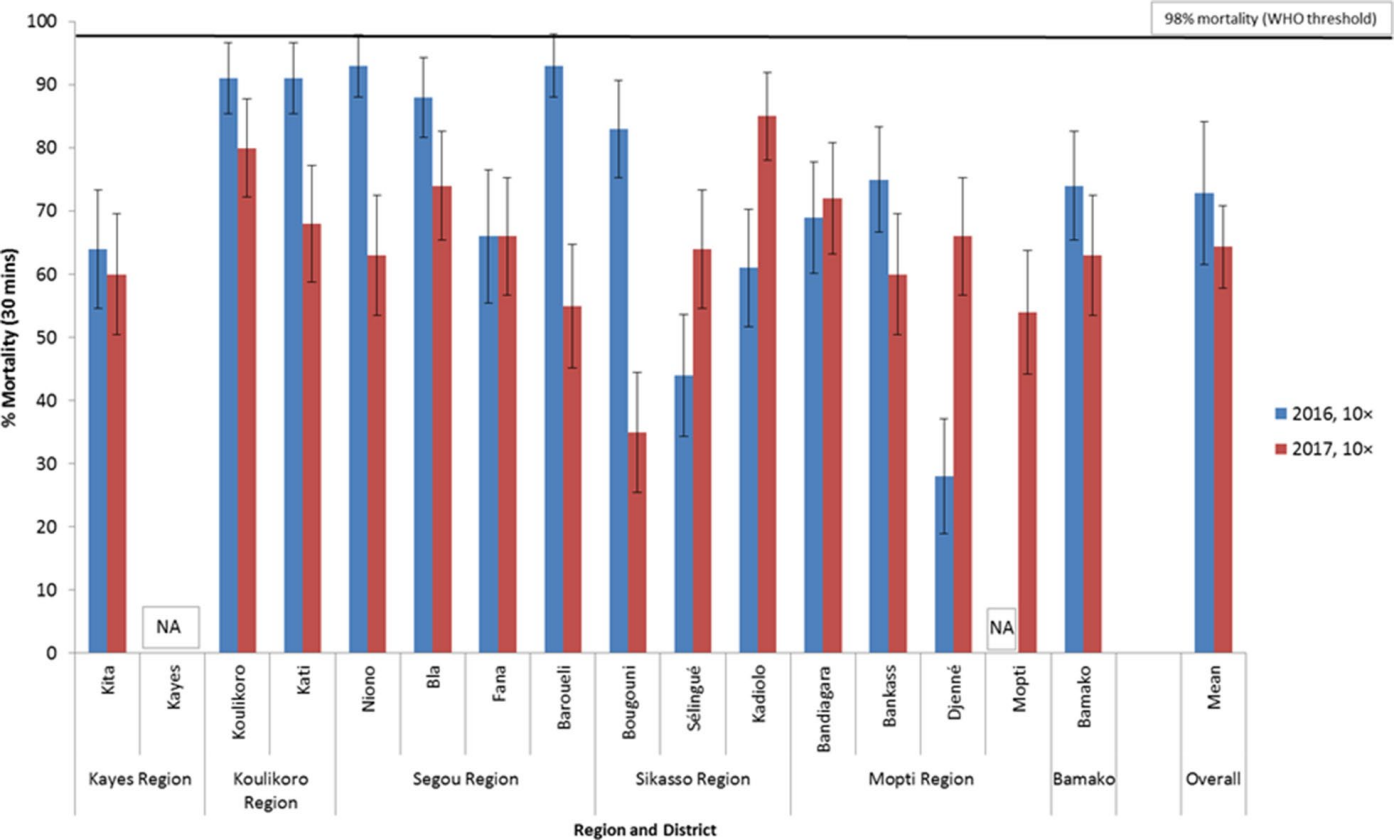
Percent mortality of *An. gambiae* (*s.l*.) after 30 min exposure to 10× the diagnostic concentration of permethrin (215 µg ai/bottle) in CDC bottle bioassays in 2016 and 2017. *Abbreviation*: NA, no data

**Fig. 3:**
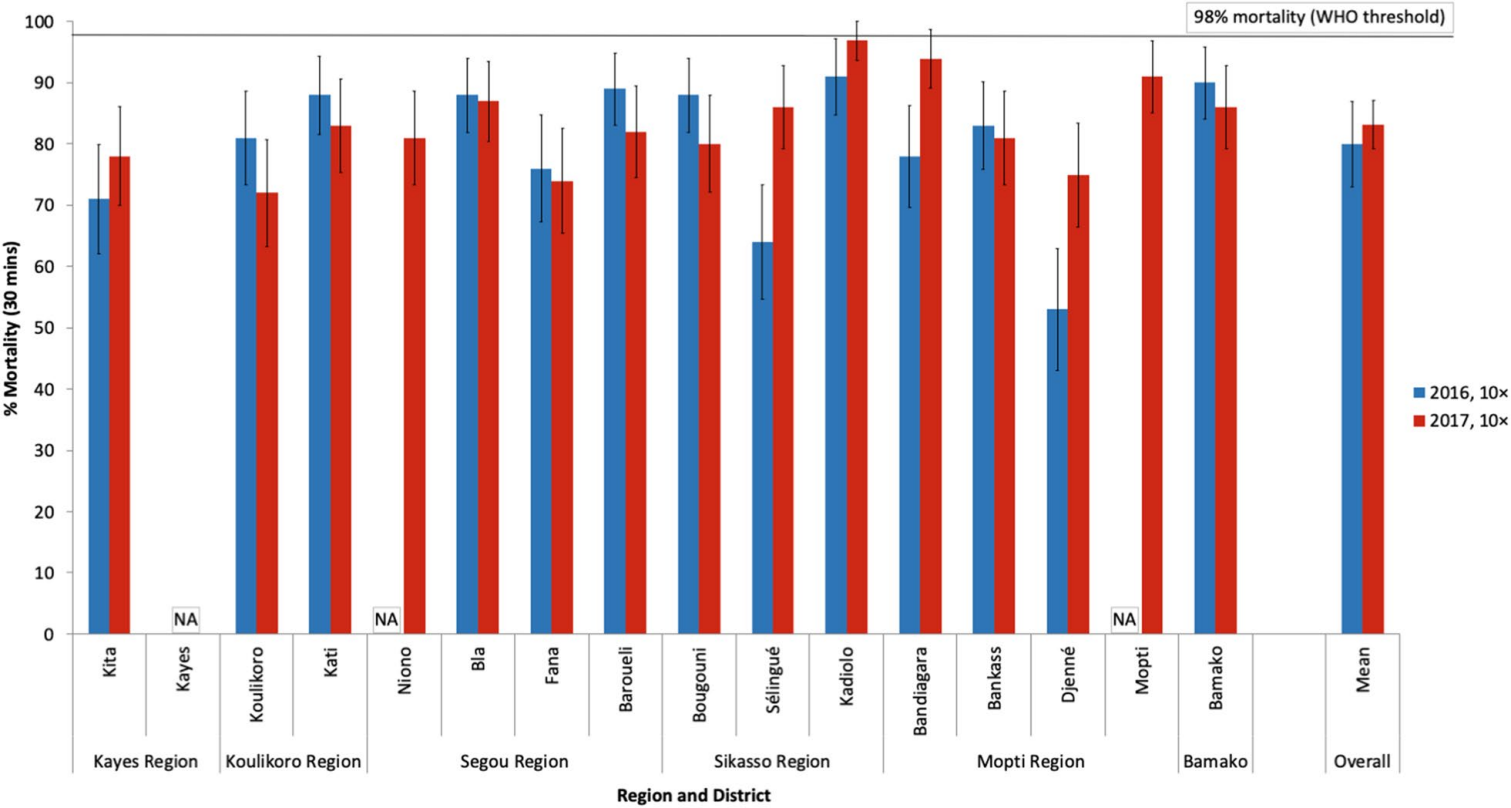
Percent mortality of *An. gambiae* (*s.l*.) after 30 min exposure to 10× the diagnostic concentration of deltamethrin (125 µg ai/bottle) in bottle bioassays in 2016 and 2017. *Abbreviation*: NA, no data

Figure 2 shows mortality rates of *An. gambiae* following 30 min exposure to permethrin 10× **(**215 µg ai/bottle) and Fig. 3 for deltamethrin 10× (125 µg (ai)/bottle). Additional data are provided showing results of 1×, 2× and 5× in Additional file 1: Table S1. In all sites *An. gambiae* (*s.l*.) showed high intensity resistance to permethrin and deltamethrin in 2016 and 2017. Overall, mortality rates to permethrin 10× ranged from 28% to 93% in 2016 and from 35% to 85% in 2017 (Fig. 2). With deltamethrin 10×, mortality rates varied from 53% to 91% in 2016 and from 72% to 97% in 2017 (Fig. 3). Mean trends showed there may have been a slight increase in permethrin resistance intensity in 2017 compared to 2016, but for deltamethrin trends were similar in 2016 and 2017.

### *An. gambiae* (*s.l*.) intensity of resistance to alpha-cypermethrin, permethrin and deltamethrin in WHO tube tests in 2018

**Fig. 4:**
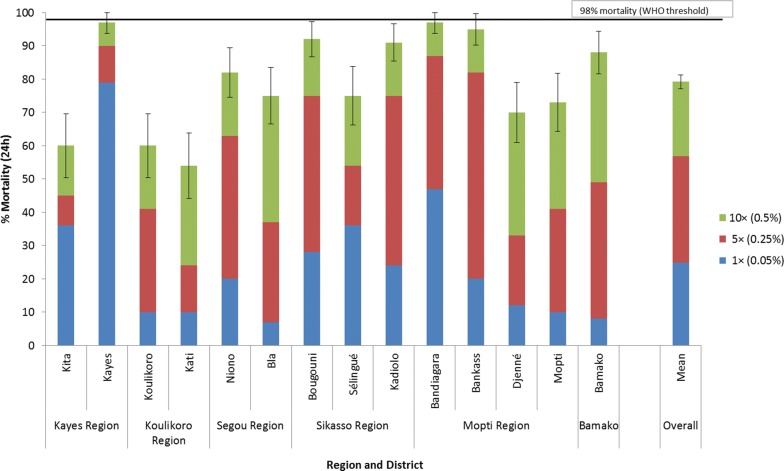
Percent mortality of *An. gambiae* (*s.l*.) tested in WHO tube tests using 1× (0.05%), 5× (0.25%) and 10× (0.50%) the diagnostic concentration of alpha-cypermethrin in 2018

**Fig. 5:**
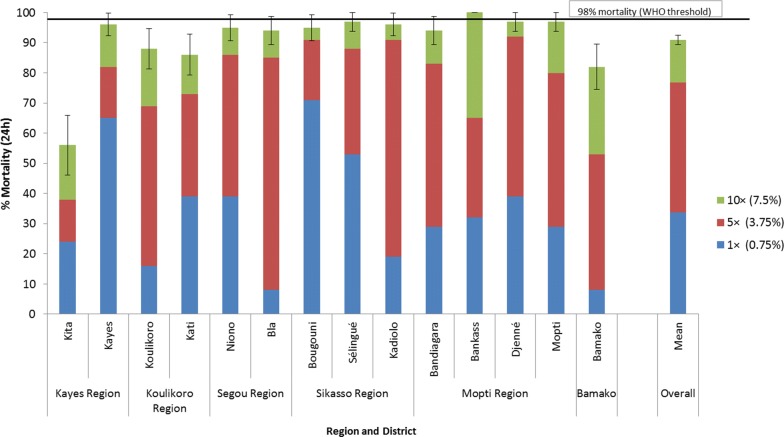
Percent mortality of *An. gambiae* (*s.l*.) tested in WHO tube tests using 1× (0.75%), 5× (3.75%) and 10× (7.5%) the diagnostic concentration of permethrin in 2018

**Fig. 6:**
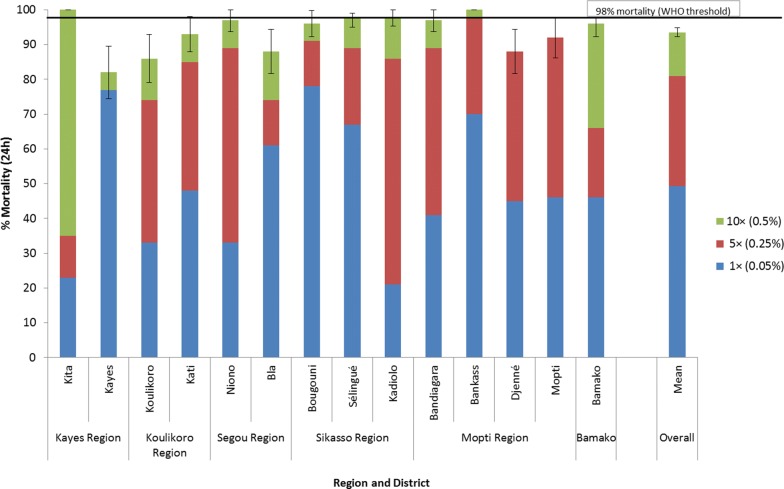
Percent mortality of *An. gambiae* (*s.l*.) tested in WHO tubes using 1× (0.05%), 5× (0.25%) and 10× (0.5%) the diagnostic concentration of deltamethrin in 2018

Figures 4, 5, 6 show resistance intensity results for *An. gambiae* (*s.l*.) against alpha-cypermethrin, permethrin, and deltamethrin at 1×, 5× and 10× the WHO diagnostic concentration. High intensity resistance was recorded in all sites to alpha-cypermethrin (mortality < 98% at 10× dose) (Fig. 4). The mean percent mortality across all sites for alpha-cypermethrin was 24.8% at 1×, 56.9% at 5× and 79.2% at 10× the diagnostic concentration.

Resistance intensity was also high in most sites to permethrin and deltamethrin (Figs. 5 and 6). The mean percent mortality across all sites for permethrin was 33.6% at 1×, 76.9% at 5× and 90.9% at 10×. For deltamethrin the mean percent mortality was 49.2% at 1×, 80.9% at 5× and 93.5% at 10×.

### Synergist assays using piperonyl-butoxide (PBO) and pyrethroids

**Fig. 7:**
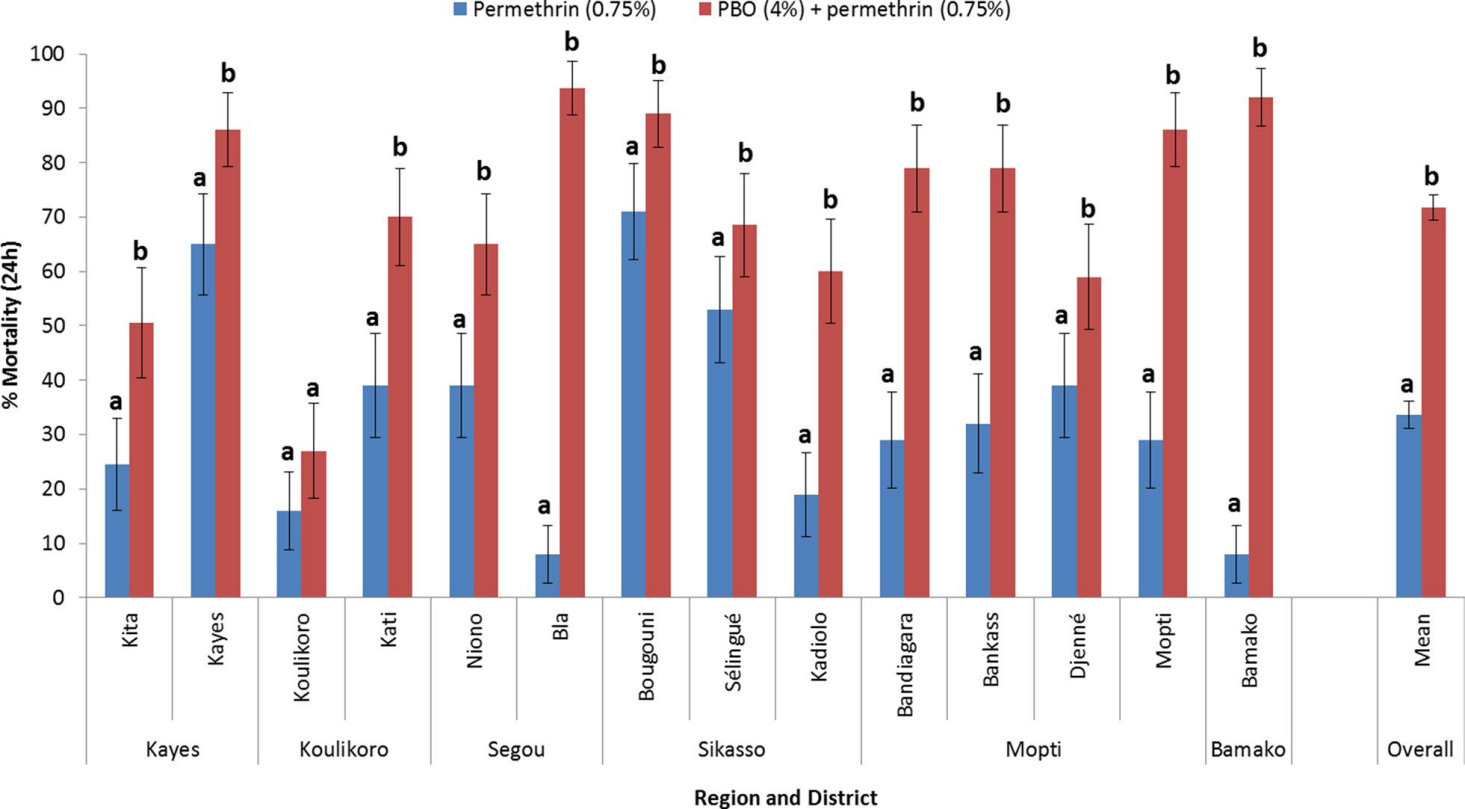
Percent mortality (24 h) of *An. gambiae* (*s.l*.) tested with permethrin (0.75%) alone and after pre-exposure to PBO (4%) synergist using WHO tube tests (bars for the same site sharing the same superscript letter (a or b) do not differ significantly, *P* > 0.05)

**Fig. 8:**
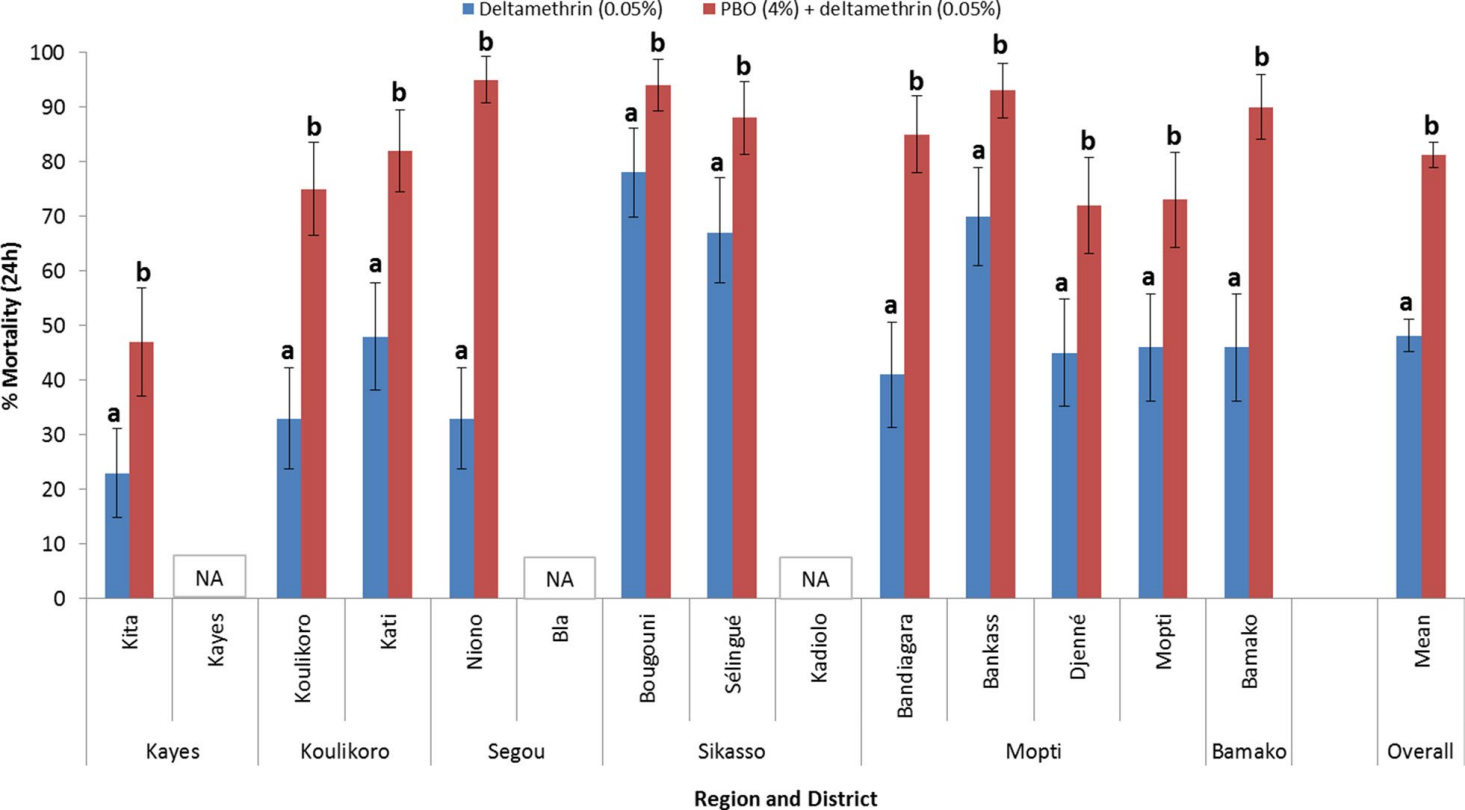
Percent mortality (24 h) of *An. gambiae* (*s.l*.) tested with deltamethrin (0.05%) alone and after pre-exposure to PBO (4%) synergist using WHO tube tests (bars for the same site sharing the same superscript letter (a or b) do not differ significantly, *P* > 0.05). *Abbreviation*: NA, no data

Results in Figs. 7 and 8 show that overexpression of mixed function oxidases (MFOs) is an important resistance mechanism in Mali, as shown by significantly greater mortality rates after PBO pre-exposure. Figure 7 shows that pre-exposure to PBO resulted in significantly greater mortality than for permethrin alone, in 13 of 14 sites (*P* < 0.05), with Koulikoro the only site where there was no apparent response to PBO. Across all sites mean mortality was 33.7% with permethrin compared with 71.8% when pre-exposed to PBO, representing a 2.13-fold increase in mortality (*P* < 0.0001).

Figure 8 shows that for all 11 sites there was a significant increase in mortality caused by deltamethrin following PBO pre-exposure. Although significantly increased mortality rates were obtained in nearly all sites for both insecticides after pre-exposure of *An. gambiae* (*s.l*.) to PBO, susceptibility was not fully restored in any sites. Mortality levels did increase to > 90% in two sites with PBO + permethrin (Bamako and Bla) and four sites (Niono, Bougouni, Bankass and Bamako) with PBO + deltamethrin. Across all sites mean mortality was 48.2% with deltamethrin compared with 81.3% when pre-exposed to PBO, representing a 1.69-fold increase in mortality (*P* < 0.0001).

### Susceptibility of *An. gambiae* (*s.l*.) to pirimiphos-methyl (0.25%) in WHO susceptibility tube tests in 2016, 2017 and 2018

In all years, susceptibility (mortality rate ≥ 98%) to pirimiphos-methyl (0.25%) was observed in all sites where pirimiphos-methyl CS has previously been sprayed for malaria control, including Koulikoro, Baroueli, Djenné, Bandiagara and Bankass. Susceptibility was also recorded in all other sites, except for Selingue, where possible resistance was noted in 2018 (96.7% mortality) (Additional file 1: Table S2).

### Susceptibility of *An. gambiae* (*s.l*.) to bendiocarb (0.1%) in WHO susceptibility tube tests in 2016, 2017 and 2018

In 2016, susceptibility (mortality rate ≥ 98%) was obtained with bendiocarb (0.1%) in all sites except for Niono (95%) and Bougouni (92%), where possible resistance was observed. In 2017, susceptibility was observed in 5 sites (Fana, Koulikoro, Bla, Djenné and Bankass), with possible resistance (90–97% mortality) in 4 sites (Kita, Kati, Bamako and Bandiagara) and resistance (< 90%) in 3 sites (Barouéli, Sélingué and Bougouni). In 2018, susceptibility was noted in all 6 sites where testing was conducted (Bla, Selingue, Bougouni, Djenné, Bandiagara and Bankass). Mortality rates to bendiocarb in the surveyed sites are summarized in Additional file 1: Table S3.

### Susceptibility of *An. gambiae* (*s.l*.) to chlorfenapyr (100 µg ai/bottle) in CDC bottle bioassays

**Fig. 9:**
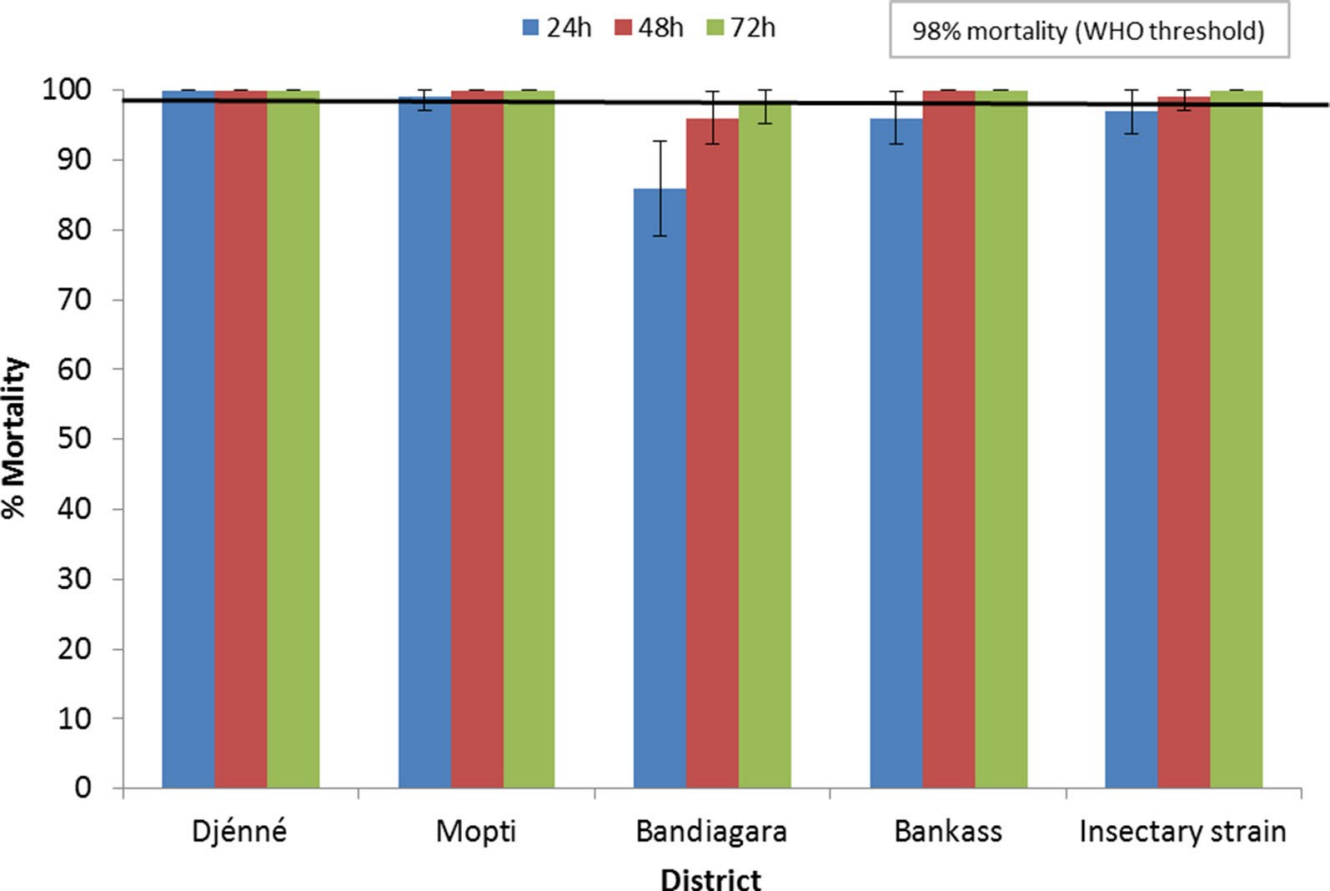
Results of *An. gambiae* (*s.l*.) (field-collected as larvae) and *An. coluzzii* Ngousso (susceptible insectary strain) susceptibility tests against chlorfenapyr (100 µg ai/bottle) in 2017

Figure 9 displays mortality rates obtained 24, 48 and 72 h after exposing *An. gambiae* (*s.l*.) from Djenné, Mopti, Bandiagara and Bankass to chlorfenapyr at 100 µg ai/bottle in 2017. After 48 h, susceptibility (mortality rate ≥ 98%) with both field-collected *An. gambiae s.l.* and insectary *An. coluzzii* Ngousso (susceptible insectary strain) was determined at all sites, except Bandiagara. *Anopheles gambiae* (*s.l*.) mortality was < 98% in Bandiagara after 48 h but did reach 98% at 72 h (the diagnostic time). Therefore, susceptibility to chlorfenapyr was recorded in all sites within 72 h of exposure.

### Susceptibility of *An. gambiae* (*s.l.*) to clothianidin (2%) in WHO susceptibility tube tests in 2018

**Fig. 10:**
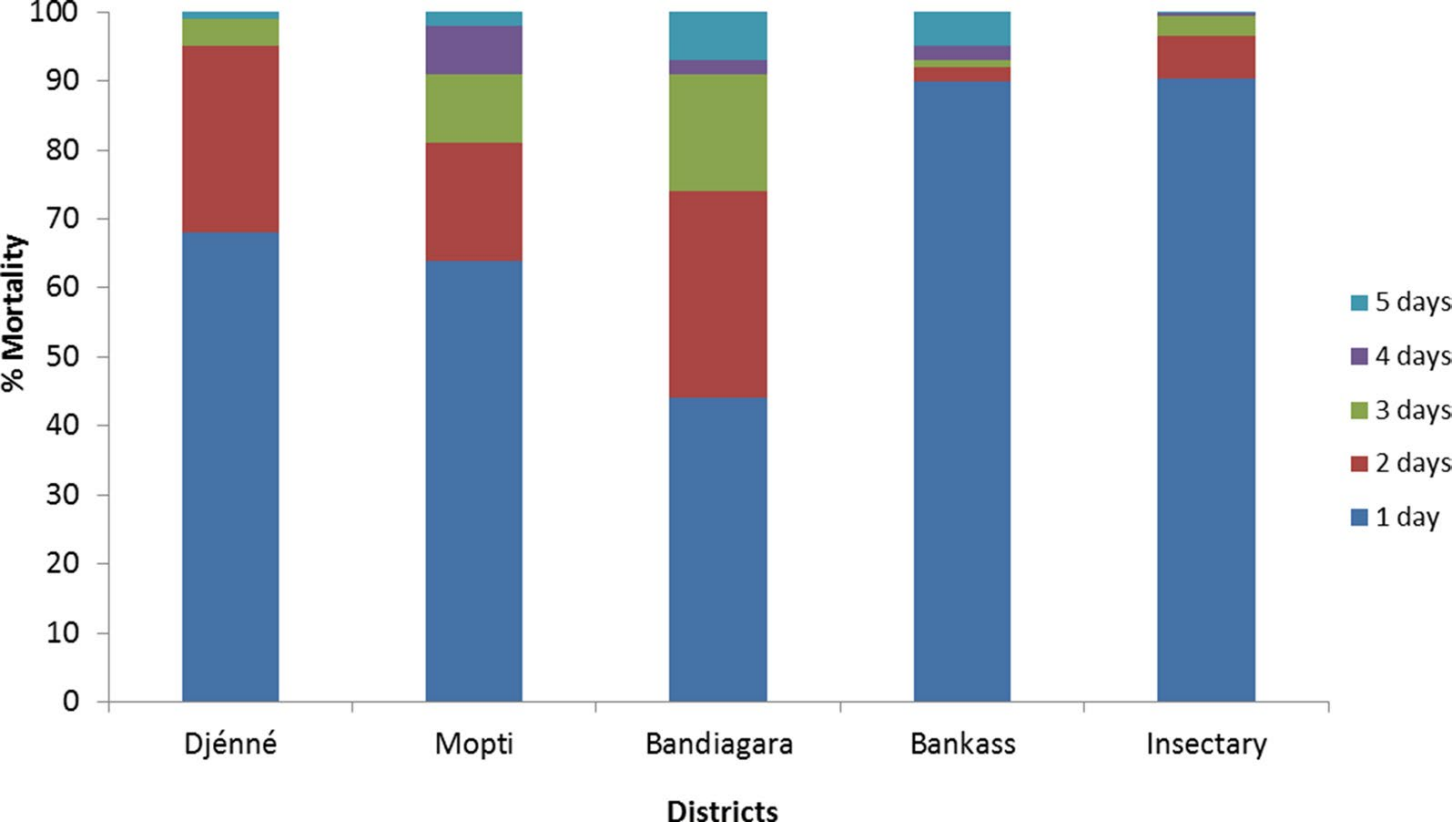
Mortality of *An. gambiae* (*s.l*.) (collected as larvae) from four IRS sites tested against clothianidin 2% in WHO tube tests in 2018

Figure 10 shows mortality rates following exposure to clothianidin 2% of *An. gambiae* (*s.l*.) (collected as larvae) from four IRS sites (Djenné, Mopti, Bandiagara and Bankass). Parallel tests were conducted with the same papers using the susceptible insectary strain of *An. coluzzii* Ngousso. Twenty-four hours after exposure, mortality rates were 90% for the insectary strain and between 44–90% for wild *An. gambiae* (*s.l*.). For the insectary strain, 99% mortality was observed three days after exposure, with mortality rates being slightly lower for wild *An. gambiae* (*s.l*.). One hundred percent mortality was recorded for insectary and wild *An. gambiae* (*s.l*.), five days after exposure, indicating susceptibility to clothianidin in all four IRS sites. Mortality rates in negative controls were low and varied from 0% to 10% after five days.

### Comparison of CDC bottle bioassays and WHO susceptibility tube tests for determining pyrethroid resistance intensity

**Fig. 11:**
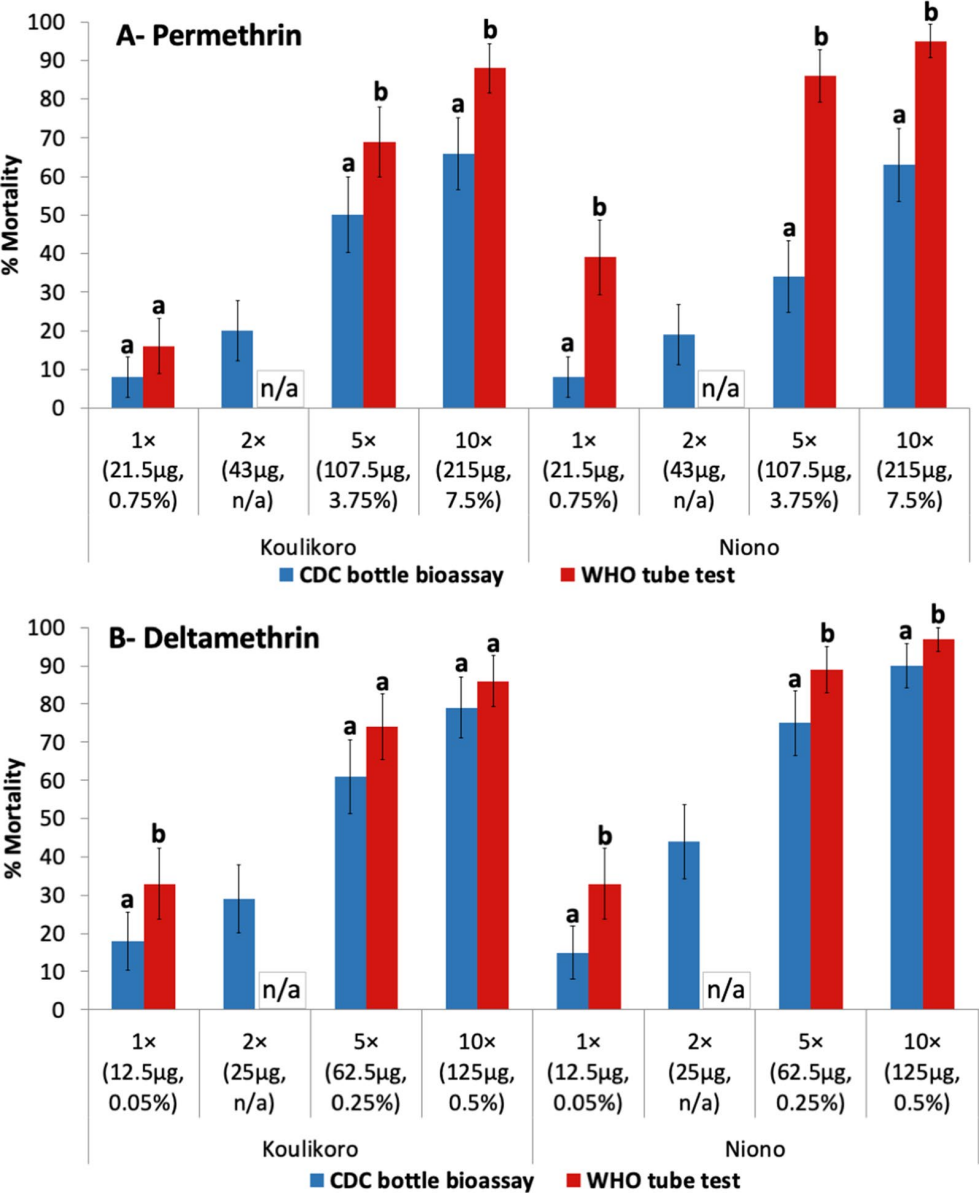
Percent mortality of *An. gambiae* (*s.l*.) in WHO tube test (24 h mortality) and CDC bottle bioassay (30 min mortality) to permethrin and deltamethrin in Koulikoro and Niono. Statistical comparison was made comparing results of WHO tube tests and CDC bottle bioassay by site and dose tested (bars for the same site sharing the same superscript letter (a or b) do not differ significantly, *P* > 0.05). *Abbreviation*: NA, no data (2× dose not tested for WHO tube test)

Figure 11 shows percentage mortality of *An. gambiae* (*s.l*.) to permethrin and deltamethrin at doses of 1×, 2×, 5× and 10× the diagnostic concentration, using both bottle bioassays (30 min mortality) and WHO susceptibility tube tests (24 h mortality) in Koulikoro and Niono in 2018. Both methods indicate high intensity pyrethroid resistance in Koulikoro and Niono (mortality < 98% at 10×). Testing conducted in Niono consistently produced higher mortality rates for both permethrin and deltamethrin with WHO tube tests as compared to CDC bottle bioassays at all doses (*P* < 0.05). In Koulikoro, the 5× and 10× doses of permethrin produced higher mortality for permethrin in WHO tube tests than CDC bottle bioassay. However, there was only a difference at the 1× dose with deltamethrin in Koulikoro.

### Molecular species identification of the *An. gambiae* species complex

**Fig. 12:**
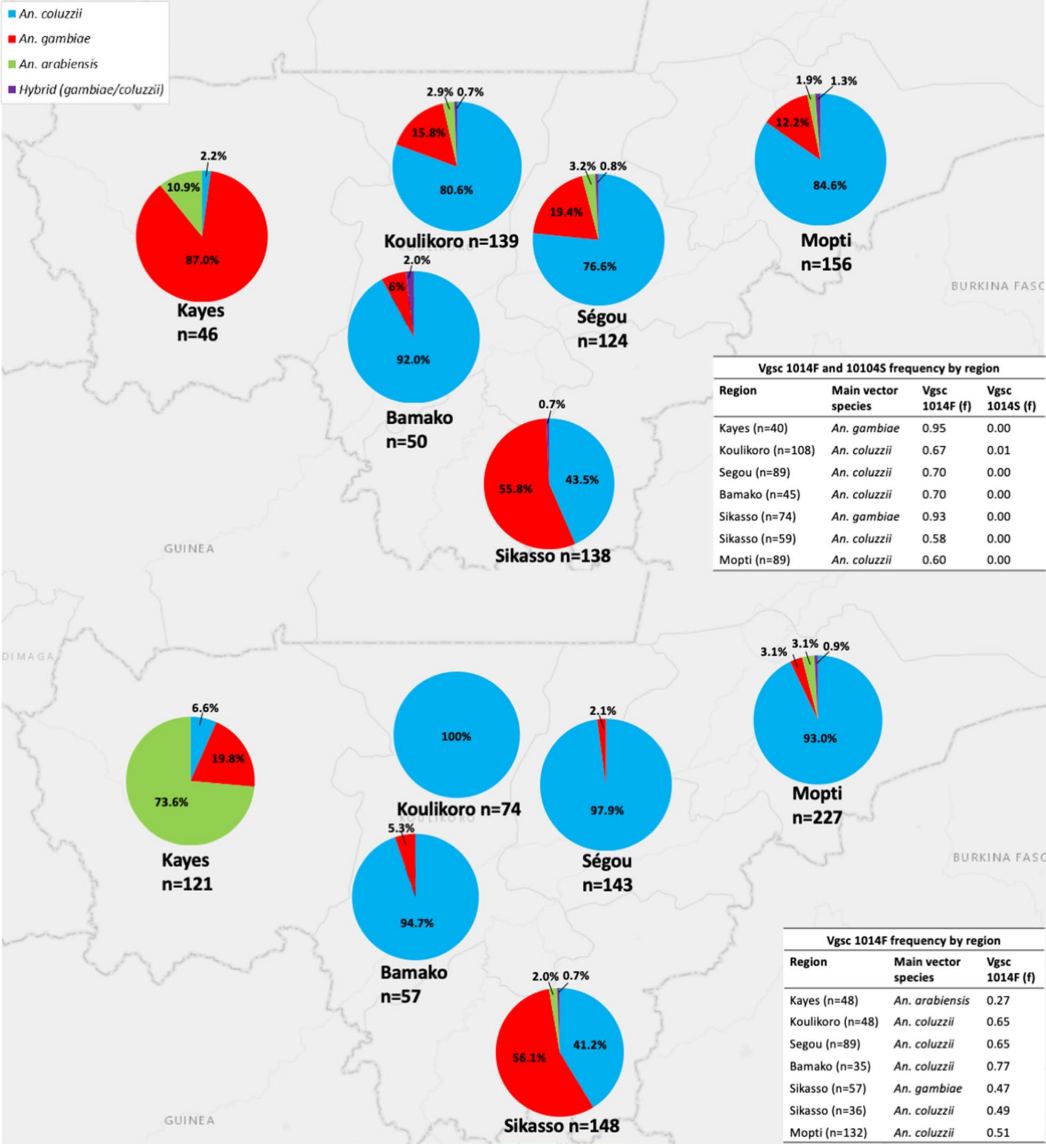
*An. gambiae* (*s.l*.) sibling species composition and *vgsc*-1014F and *vgsc*-1014S frequency in the six surveyed regions in 2017 (top) and 2018 (bottom)

In 2017 and 2018, adult *An. gambiae* (*s.l*.) specimens (collected as larvae) used for susceptibility tests from sentinel sites (*c.*50 mosquitoes/site) located in 6 regions (Fig. 1), were tested by PCR for species identification. Figure 12  summarizes *An. gambiae* (*s.l*.) sibling species composition by region and L1014F/S frequency. *Anopheles coluzzii* was the primary vector in 4 regions (Koulikoro, Ségou, Mopti and Bamako) in 2017 and 2018. In the southern region of Sikasso, slightly more than half of the mosquitoes were *An. gambiae*, with just over 40% being *An. coluzzii* in both years. Some hybrid samples (*An. gambiae × An. coluzzii*) were recorded at low frequency (≤ 2% by region).

In the Kayes Region, the composition changed from predominantly *An. gambiae* in 2017 to *An. arabiensis* in 2018. However, the sites in the region changed during this period with Kita maintained in both years and the western Kayes site (Fig. 1) only surveyed during 2018 (which accounted for most *An. arabiensis*). *Anopheles arabiensis* was present at a relatively low frequency in all other regions (2–10%). Between 3*–*24% of samples did not amplify by PCR using primers of the *An. gambiae* (*s.l*.) complex. Samples may have failed to amplify due to degraded DNA or due to morphological misidentification (i.e. non-*An. gambiae* (*s.l*.)).

The frequencies of *vgsc*-1014F and 1014S alleles are summarized in Fig. 12 for the main vector species of each region. In 2017, the *vgsc*-1014S allele was absent in most regions, only being detected at a frequency of 0.01 in Koulikoro. Testing of the *vgsc*-1014S allele was not conducted in 2018. The *vgsc-*1014F allele was present at moderate to high frequency in all regions during 2017 and 2018 for *An. gambiae* (0.47–0.95) and *An. coluzzii* (0.58–0.77) but was at low frequency for *An. arabiensis* (0.27).

## Discussion

Pyrethroid resistance has been widespread in Mali for several years. A study by Cisse et al. in 2012 has shown that *An. gambiae* (*s.l*.) were resistant to lambda-cyhalothrin in all nine sites tested and to deltamethrin in three of four sites [9]. The *vgsc*-1014F mutation, which is associated with pyrethroid resistance, has been present in *An. gambiae* (*s.l*.) in Mali since 1987 (albeit at low frequency) [20]. Fanello et al. [20] have shown that *vgsc*-1014F increased in frequency in Banambani (Koulikoro Region) from 3% in 1987 to 62% in 2000, presumably due to an increase in selection pressure from agriculture and early insecticide-treated net (ITN) use. The gradual increase in *vgsc*-1014F frequency in Mali was also reported by Tripet et al. [21].

Although pyrethroid resistance has been present for more than 20 years in malaria vectors throughout Mali [20], it is not clear to what extent LLIN efficacy has been compromised. Numerous small-scale studies in sub-Saharan Africa have demonstrated reduced control of resistant malaria vectors by pyrethroid LLINs [8, 22, 23]. A multi-country evaluation coordinated by the WHO provided evidence that LLINs continue to provide some degree of personal protection against malaria in areas with pyrethroid resistance, but did not monitor community impact caused by the mosquito killing effect of LLINs [24]. The World Malaria Report (2018) [25] noted that global progress against malaria has stalled, with no significant progress in reducing global malaria cases between 2015–2017, with a possible explanation being the widespread occurrence of pyrethroid-resistant malaria vectors.

The concept of resistance intensity is relatively new, having first been included in the WHO testing guidelines in 2016 [13]. Nevertheless, high intensity pyrethroid resistance is being reported in an increasing number of locations, including Accra in Ghana [26], Lagos and Ogun in Nigeria [27], western Kenya [28] and south-western Burkina Faso [29]. Despite uncertainty regarding the impact of pyrethroid resistance, the WHO states that “when resistance is confirmed at the 5× and especially at the 10× concentrations, operational failure is likely” [13]. Throughout Mali, resistance to the three most common pyrethroids used on LLINs was confirmed at the 5× and 10× concentrations, therefore making it highly likely that pyrethroid LLINs are no longer providing optimal protection against malaria. High intensity pyrethroid resistance appears to be present in all three major malaria vector species in Mali, with *An. arabiensis* in the Kayes region, *An. gambiae* (*s.s*.) in the Sikasso region and *An. coluzzii* in Koulikoro, Segou, Bamako and Mopti regions exhibiting high intensity resistance to permethrin, deltamethrin and alpha-cypermethrin.

In Koulikoro and Niono, where CDC bottle bioassays as well as WHO susceptibility tube tests were used simultaneously, high intensity pyrethroid resistance was observed for both methods. However, in most cases, significantly higher mortality rates were observed in WHO tube tests than in CDC bottle bioassays. Owusu et al. [30] concluded that the two assays can both successfully detect insecticide resistance, but there was a high level of inconsistency between the two methods when using the diagnostic concentration. There are pros and cons to both methods for determining resistance intensity. In this study, a decision was made to switch from CDC bottle bioassays to WHO papers due to the ease of use and standardized provision of treated filter papers from WHO, compared to self-treatment of bottles which may result in more technician-induced test variation.

Unlike in previous years, there are an increasing number of LLIN options for the control of pyrethroid resistant malaria vectors, including several brands of PBO synergist nets (involving mixtures of PBO plus permethrin, deltamethrin or alpha-cypermethrin). There are also LLINs that contain new insecticides for malaria vector control, such as Interceptor G2® (BASF, Ludwigshafen, Germany), a mixture of chlorfenapyr (pyrrole) and alpha-cypermethrin (pyrethroid), while Olyset Duo® (Sumitomo Chemical Company, Tokyo, Japan) and Royal Guard® (Disease Control Technologies LLC, Greer, USA), are both mixtures of pyriproxyfen (juvenile hormone mimic) plus a pyrethroid. The main factors limiting the uptake of these alternative products are the increased cost and a lack of epidemiological evidence to show improved performance over pyrethroid nets. Preliminary results show susceptibility to chlorfenapyr in Mali. As part of the ‘New Nets Project’, two million Interceptor G2 nets are being distributed in several countries, including Mali in 2020 as an operational pilot to build evidence regarding the cost-effectiveness of dual active ingredient nets [31].

Results of synergist bioassays in Mali indicated that metabolic resistance is present nationwide and pre-exposure to PBO significantly increased pyrethroid-induced mortality. Full susceptibility was not recovered using PBO, indicating a combination of resistance mechanisms, with moderate or high *vgsc*-1014F frequencies in *An. coluzzii* and *An. gambiae*. In Tanzania, use of PBO LLINs resulted in reduced malaria incidence compared to standard pyrethroid LLINs [32]. However, it is not clear what level of mortality is required in PBO bioassays to result in epidemiological impact as there was no bioassay data in the Tanzania study. Synergist bioassays in Mali suggest that PBO LLINs should provide better control than pyrethroid LLINs, although the epidemiological impact is uncertain. A previous published study by Cisse et al. [33] appeared to show no benefit of PBO LLINs in Bougouni, Mali. However, this study was conducted in 2014 in a location where synergist bioassays showed much lower metabolic resistance levels [9]. A study by Protopopoff et al. [32] in Tanzania showed that PBO LLINs improved control of malaria transmission compared to standard pyrethroid LLINs, but there was no additional benefit of non-pyrethroid IRS combined with PBO LLINs when compared to PBO LLINs alone. While more epidemiological studies are needed, this finding suggests that PBO and next generation LLINs should be prioritized for non-IRS areas.

Pyrethroid insecticides have not been used for IRS in Mali since 2009, when resistance was detected. The good news is that there are viable options for IRS in Mali, with susceptibility recorded to clothianidin and pirimiphos-methyl. The PMI VectorLink Programme is currently implementing IRS in the Mopti Region of Mali with these products in rotation for resistance management.

## Conclusions

High intensity pyrethroid resistance is widespread in Mali and threatens the efficacy of pyrethroid LLINs. Synergist bioassays suggest that PBO LLINs should provide improved control in most districts of Mali. *Anopheles gambiae* (*s.l*.) was susceptibile to chlorfenapyr, indicating that next-generation LLINs are also a viable alternative. Susceptibility to clothianidin and pirimiphos-methyl was confirmed, with both insecticides currently being used for IRS as part of a rotation strategy for resistance management.

## Supplementary information

**Additional file 1: Table S1.** Percentage mortality of *An. gambiae* (*s.l*.) tested with permethrin and deltamethrin in 2016 and 2017 at 1×, 5× and 10× the diagnostic concentraionin CDC bottle intensity bioassays. **Table S2.** Mortality of *An. gambiae* (*s.l*.) tested with 0.25% pirimiphos-methyl in 2016, 2017 and 2018. *Abbreviations*: S, susceptible; PR: possible resistance; R, resistant. **Table S3.** Mortality of *An. gambiae* (*s.l*.) tested with 0.1% bendiocarb in 2016, 2017 and 2018. *Abbreviations*: S, Susceptible; PR, possible resistance; R, resistant.

## Data Availability

The datasets used and/or analyzed during the current study are available from the corresponding author upon reasonable request.

## Abbreviations

AI: Active ingredient
BASF: Badische Anilin und Soda Fabrik
CDC: United States Centers for Disease Control and Prevention
CS: Capsule suspension
DHS: Demographic and health survey
IRS: Indoor residual spraying
ITN: Insecticide-treated net
LLIN: Long-lasting insecticidal net
NMCP: National Malaria Control Programme
PBO: Piperonyl butoxide
PCR: Polymerase chain reaction
PMI: United States President’s Malaria Initiative
TGAI: Technical grade active ingredient
SCC: Sumitomo Chemical Company
VGSC: Voltage gated sodium channel
WHO: World Health Organization
WG: Water dispersible granules
WP: Wettable powder
