# Supplementary material for: Anopheles gambiae (s.l.) exhibit high intensity pyrethroid resistance throughout Southern and Central Mali (2016–2018): PBO or next generation LLINs may provide greater control
Source: Parasit Vectors. 2020 May 8;13:239. doi: 10.1186/s13071-020-04100-7 (PMC7206711; doi:10.1186/s13071-020-04100-7)
Supplement: Supplementary file 1 — Additional file 1: Table S1. Percentage mortality of An. gambiae (s.l.) tested with permethrin and deltamethrin in 2016 and 2017 at 1×, 5× and 10× the diagnostic concentraionin CDC bottle intensity bioassays. Table S2. Mortality of An. gambiae (s.l.) tested with 0.25% pirimiphos-methyl in 2016, 2017 and 2018. Abbreviations: S, susceptible; PR: possible resistance; R, resistant. Table S3. Mortality of An. gambiae (s.l.) tested with 0.1% bendiocarb in 2016, 2017 and 2018. Abbreviations: S, Susceptible; PR, possible resistance; R, resistant. [file 13071_2020_4100_MOESM1_ESM.docx]

Additional file 1: Table S1. Percentage mortality of *An. gambiae* (*s.l*.) tested with permethrin and deltamethrin in 2016 and 2017 at 1×, 5× and 10× the diagnostic concentraion in CDC bottle intensity bioassays.

| **Sites** | **Permethrin 2016** | | | | **Permethrin 2017** | | | | **Deltamethrin 2016** | | | | **Deltamethrin 2017** | | | |
| --- | --- | --- | --- | --- | --- | --- | --- | --- | --- | --- | --- | --- | --- | --- | --- | --- |
|  | **1X** | **5X** | **10X** | **Intensity of resistance** | **1X** | **5X** | **10X** | **Intensity of resistance** | **1X** | **5X** | **10X** | **Intensity of resistance** | **1X** | **5X** | **10X** | **Intensity of resistance** |
| **Djénné** | 17 | 27 | 28 | High | 05 | 38 | 66 | High | 38 | 60 | 53 | High | 11 | 71 | 75 | High |
| **Mopti** | - | - | - | - | 06 | 36 | 54 | High | - | - | - | - | 29 | 83 | 91 | High |
| **Bandiagara** | 19 | 49 | 69 | High | 26 | 58 | 72 | High | 38 | 87 | 78 | High | 41 | 69 | 94 | High |
| **Bankass** | 42 | 81 | 75 | High | 02 | 40 | 60 | High | 43 | 74 | 83 | High | 22 | 72 | 81 | High |
| **Koulikoro** | 34 | 92 | 91 | High | 06 | 59 | 80 | High | 2 | 84 | 81 | High | 26 | 55 | 72 | High |
| **Fana** | 18 | 36 | 66 | High | 12 | 55 | 66 | High | 21 | 48 | 76 | High | 36 | 70 | 74 | High |
| **Baroueli** | 03 | 30 | 93 | High | 10 | 17 | 55 | High | 39 | 71 | 89 | High | 12 | 65 | 82 | High |
| **Bamako** | 34 | 36 | 74 | High | 00 | 47 | 63 | High | 84 | 80 | 90 | High | 7 | 54 | 86 | High |
| **Kati** | 78 | 86 | 91 | High | 06 | 40 | 68 | High | 84 | 61 | 88 | High | 26 | 61 | 83 | High |
| **Bla** | 20 | 88 | 88 | High | 12 | 68 | 74 | High | 64 | 74 | 88 | High | 18 | 76 | 87 | High |
| **Selingue** | 04 | 29 | 44 | High | 05 | 24 | 64 | High | 23 | 51 | 64 | High | 11 | 43 | 86 | High |
| **Bougouni** | 09 | 17 | 83 | High | 16 | 28 | 35 | High | 43 | 92 | 88 | High | 14 | 35 | 80 | High |
| **Niono** | 16 | - | 93 | High | 05 | 43 | 63 | High | - | - | - | - | 26 | 69 | 81 | High |
| **Kita** | 47 | 64 | 64 | High | 04 | 35 | 60 | High | 62 | 77 | 71 | High | 11 | 58 | 78 | High |
| **Kadiolo** | 07 | 43 | 61 | High | 06 | 71 | 85 | High | 55 | 90 | 91 | High | 74 | 87 | 97 | High |
| **Mean** | **21** | **52** | **73** | **High** | **08** | **44** | **64** | **High** | **46** | **73** | **80** | **High** | **24** | **65** | **83** | **High** |

**Additional file 1: Table S2. Mortality of *An. gambiae* (*s.l*.)** **tested with 0.25% pirimiphos-methyl in 2016, 2017 & 2018.**

|  | Pirimiphos-methyl (0.25%) | | | | | | | |
| --- | --- | --- | --- | --- | --- | --- | --- | --- |
|  | 2016 | |  | 2017 | |  | 2018 | |
| Sites | Total tested | Mortality (%) _  Resistance Status |  | Total tested | Mortality (%) _  Resistance Status |  | Total tested | Mortality (%) _  Resistance Status |
|  |  |  |  |  |  |  |  |  |
| Kita | 100 | 100 _S |  | 97 | 100 _S |  | 94 | 100 _S |
| Fana | 100 | 100 _S |  | 97 | 100 _S |  | - | - |
| Koulikoro | 103 | 100 _S |  | 100 | 100 _S |  | 92 | 100 _S |
| Kati | 100 | 100 _S |  | 102 | 100 _S |  | 100 | 98 _S |
| Bamako | 100 | 100 _S |  | 100 | 100 _S |  | 100 | 100 _S |
| Bla | 100 | 99 _S |  | 100 | 100 _S |  | 100 | 100 _S |
| Baroueli | 100 | 100 _S |  | 100 | 100 _S |  | - | - |
| Niono | - | - |  | - | - |  | - | - |
| Selingue | 100 | 100 _S |  | 100 | 100 _S |  | 91 | 96.7 _PR |
| Bougouni | 81 | 100 _S |  | 100 | 100 _S |  | 100 | 100 _S |
| Kadiolo | 100 | 100 _S |  | - | - |  | 100 | 99 _S |
| Djenné | 94 | 100 _S |  | 100 | 100 _S |  | 100 | 100 _S |
| Bandiagara | 104 | 100 _S |  | 100 | 100 _S |  | 100 | 100 _S |
| Bankass | 104 | 100 _S |  | 102 | 100 _S |  | 100 | 100 _S |
| Total | 1286 | 99.9 _S |  | 1198 | 100 _S |  | 1077 | 99.4 _S |

S: Susceptible, PR: Possible Resistance, R: Resistant

**Additional file 1: Table S3. Mortality of *An. gambiae* (*s.l*.) tested with 0.1% Bendiocarb in 2016, 2017& 2018**

|  | Bendiocarb (0.1%) | | | | | | | | | | |
| --- | --- | --- | --- | --- | --- | --- | --- | --- | --- | --- | --- |
|  | 2016 | | |  | 2017 | | |  | 2018 | | |
| Sites | Total tested | Mortality (%)_  Resistance Status | CI |  | Total tested | Mortality (%) _Resistance Status | CI |  | Total tested | Mortality (%)_Resistance Status | CI |
|  |  |  |  |  |  |  |  |  |  |  |  |
| Kita | 100 | 100_S | - |  | 100 | 95_PR | [90.7-99.3] |  | - | - | - |
| Fana | 100 | 100_S | - |  | 102 | 98_S | [95.3-100] |  | - | - | - |
| Koulikoro | 104 | 99_S | [97.2-100] |  | 100 | 99_S | [97-100] |  | - | - | - |
| Kati | 100 | 98_S | [95.3-100] |  | 100 | 97_PR | [93.7-100] |  | - | - | - |
| Bamako | 100 | 98_S | [95.3-100] |  | 100 | 90_PR | [84.1-95.9] |  | - | - | - |
| Bla | 100 | 100_S | - |  | 100 | 98_S | [95.3-100] |  | 100 | 100_S | - |
| Baroueli | 100 | 98_S | [95.3-100] |  | 100 | 89_R | [82.9-95.1] |  | - | - | - |
| Niono | 75 | 95_PR | [89.6-99.8] |  | - | - | - |  | - | - | - |
| Selingue | 98 | 100_S | - |  | 99 | 86(R) | [79-92.7] |  | 80 | 100_S | - |
| Bougouni | 100 | 92_PR | [86.7-97.3] |  | 100 | 76(R) | [67.6-84.4] |  | 100 | 100_S | - |
| Kadiolo | 100 | 100_S | - |  | - | - | - |  | - | - | - |
| Djenne | 100 | 100_S | - |  | 100 | 98_S | [95.3-100] |  | 100 | 100_S | - |
| Bandiagara | 104 | 100_S | - |  | 100 | 97_PR | [93.7-100] |  | 100 | 100_S | - |
| Bankass | 104 | 100_S | - |  | 87 | 99_S | [96.6-100] |  | 100 | 100_S | - |
| Total | 1385 | 99_S | [98-99.2] |  | 1188 | 93_PR | [92-94.8] |  | 580 | 100_S | - |

S: Susceptible, PR: Possible Resistance, R: Resistant
